# Dysfunctional Interaction Between the Dorsal Attention Network and the Default Mode Network in Patients With Type 2 Diabetes Mellitus

**DOI:** 10.3389/fnhum.2021.796386

**Published:** 2021-12-24

**Authors:** Yumeng Lei, Dongsheng Zhang, Fei Qi, Jie Gao, Min Tang, Kai Ai, Xuejiao Yan, Xiaoyan Lei, Zhirong Shao, Yu Su, Xiaoling Zhang

**Affiliations:** ^1^Department of MRI, Shaanxi Provincial People’s Hospital, Xi’an, China; ^2^Department of Clinical Science, Philips Healthcare, Xi’an, China; ^3^Department of Graduate, Xi’an Medical University, Xi’an, China

**Keywords:** type 2 diabetes mellitus, anticorrelation, dorsal attention network, default mode network, functional connectivity

## Abstract

The risk of cognitive impairment in patients with type 2 diabetes mellitus (T2DM) is significantly higher than that in the general population, but the exact neurophysiological mechanism underlying this is still unclear. An abnormal change in the intrinsic anticorrelation of the dorsal attention network (DAN) and the default mode network (DMN) is thought to be the mechanism underlying cognitive deficits that occur in many psychiatric disorders, but this association has rarely been tested in T2DM. This study explored the relationship between the interaction patterns of the DAN-DMN and clinical/cognitive variables in patients with T2DM. Forty-four patients with T2DM and 47 sex-, age-, and education-matched healthy controls (HCs) underwent neuropsychological assessments, independent component analysis (ICA), and functional network connection analysis (FNC). The relationship of DAN-DMN anticorrelation with the results of a battery of neuropsychological tests was also assessed. Relative to the HC group, the DMN showed decreased functional connectivity (FC) in the right precuneus, and the DAN showed decreased FC in the left inferior parietal lobule (IPL) in patients with T2DM. Subsequent FNC analysis revealed that, compared with the HC group, the T2DM patients displayed significantly increased inter-network connectivity between the DAN and DMN. These abnormal changes were correlated with the scores of multiple neuropsychological assessments (*P* < 0.05). These findings indicate abnormal changes in the interaction patterns of the DAN-DMN may be involved in the neuropathology of attention and general cognitive dysfunction in T2DM patients.

## Introduction

Type 2 diabetes mellitus (T2DM) is an independent risk factor for Alzheimer’s disease (AD), which can lead to multiple cognitive dysfunctions, such as declining memory and attention, and seriously affect a patient’s quality of life (Karvani et al., 2019). Yet, the specific neural substrate of T2DM-related cognitive impairment remains unclear. Attention, as the initial stage of cognitive processing, is an essential basic function for the generation and performance of many cognitive processes, such as learning, memory, and executive control (Egner et al., 2018), and impairment of attention can cause a series of advanced cognitive dysfunctions (Perry et al., 2000). However, the attention control system is an endogenous basic brain neural activity, which often makes defects in the attention process difficult to detect (Katsuki and Constantinidis, 2014; Burk et al., 2018), which probably causes patients to miss the best opportunity for intervention and treatment. Studies have found that early cognitive training can slow and even reverse the process of cognitive dysfunction (Cao et al., 2016). Therefore, exploring the neural mechanism of attentional impairment in T2DM and finding biomarkers for detecting attention dysfunction will provide imaging evidence for the prevention and treatment of T2DM-related attentional impairment.

Neuroimaging studies have revealed that a reciprocal pattern of brain networks composed of functionally specialized regions in the resting-state is the neurological foundation for maintaining the complex cognitive functions of the human brain (Fox et al., 2005; Fox and Raichle, 2007). In recent years, the relationship between the dorsal attention network (DAN) and the default mode network (DMN) has become an active area of study. The DAN consists of the bilateral frontal eye fields and the parietal cortex, and it is involved in top-down processing of attentional orientation and responsible for stimulus and response preparation choices (Xia et al., 2015). The DMN is comprised of the posterior cingulate cortex (PCC), precuneus, and medial prefrontal lobe cortex, and its main function is related to the processing of internal mental activities and the monitoring and extraction of episodic memory of the internal and external environment (Whitfield-Gabrieli and Ford, 2012). The DAN and DMN exhibit an intrinsic “anticorrelation” in healthy adults, which is considered to be an essential neural substrate for flexibly allocating attentional resources (Wang et al., 2019), and it may serve as the basic connection pattern in the brain’s processing of different cognitive functions (Dixon et al., 2017); the DAN-DMN anticorrelation has been suggested to be a major contributor to normal cognitive function. More specifically, a stronger DAN-DMN anticorrelation has been associated with an index of efficient cognitive processing (Kelly et al., 2008). Moreover, a reduced anticorrelation between the two networks has been observed in a number of mental disorders, which suggests its potential as a mechanism of cognitive impairment (Qian et al., 2020; Song et al., 2021). One study found that patients with Parkinson’s Disease (PD) who had mild cognitive impairment (MCI) did not have the normal DAN-DMN anticorrelation (Baggio et al., 2015). Other studies have found that the dysfunctional anticorrelation between the DAN and DMN in MCI may have a large impact on behavioral performance, and that the dysconnectivity between the DAN and DMN might be a potential biomarker for evaluating cognitive decline in patients with AD (Wang et al., 2019). Although abnormal changes in DAN-DMN interaction have been found in a variety of cognitive-related diseases and are closely related to cognitive function, it is still unclear what the potential association is between the DAN-DMN interaction pattern and cognitive impairment in patients with T2DM.

Previous evidence has consistently indicated that T2DM involves abnormal changes in the DMN. Zhou et al. (2014) found that ALFF (amplitude of low-frequency fluctuation) values were significantly decreased in the left precuneus, cuneus and the middle frontal gyrus regions of patients with T2DM. Another study found that the PCC showed reduced functional connectivity (FC) to widespread regions of the DMN in patients with T2DM compared with the controls (Musen et al., 2012). Cui et al. (2015) found that the FC of the PCC and precuneus was significantly weakened in T2DM patients, and these functional alterations were related to the impaired visual memory, attention and psychomotor speed function. In addition, Xia et al. (2015) found that several core regions of the DAN were significantly disrupted in patients with T2DM, and disruptions in the DAN were associated with attention impairment. However, few studies have focused on these two interaction networks. Only Yang et al. (2016) has explored impaired internetwork connectivity in T2DM patients. However, probably because the sample size of that study was relatively small and the functional network was characterized based on previously defined regions, the interaction between the DAN and DMN has not been clarified. The DAN-DMN anticorrelation has robust associations with attention disorders, and it is important for maintaining the complex cognitive functions of the human brain. An abnormal interaction between the two networks is related to a disturbance of attention control and effective cognitive resource processing (Wang et al., 2019; Owens et al., 2020). Therefore, exploring the interaction patterns of the DAN and DMN based on fine spatial scales will enhance our understanding of the underlying neuropathological mechanisms of cognitive impairment, especially attentional dysfunction in T2DM.

Data-driven independent component analysis (ICA) and functional network connection analysis (FNC) methods can automatically identify meaningful brain networks and directly measure the interactions within and between multiple brain networks (Fox and Raichle, 2007; Xing et al., 2020). Therefore, ICA and FNC were used in this study to explore the FC within and between the DAN and DMN in patients with T2DM. Previous evidence has consistently suggested that a disturbance in the anticorrelation between the two intrinsic networks may be a partial reason for AD patients having difficulty concentrating, or being easily distracted (Wang et al., 2019). Many neuroimaging researchers proposed a disconnection hypothesis in AD (Delbeuck et al., 2003, 2007), and the integrity of resting-state networks (RSNs) (such as DMN and DAN) that are highly related to cognitive function are more susceptible to pathological damage (Van Dam et al., 2013; Zhu et al., 2013). The cognitive impairment of T2DM has a similar neuropathological basis as AD (Bedse et al., 2015), and the chronic hyperglycemia would accelerate the accumulation of β-amyloid and tau tangles, which promote neurodegeneration, resulting in the functional brain network damage and varying degrees of cognitive impairment (Verdile et al., 2015; Gibas, 2017). Therefore, we hypothesized that patients with T2DM would exhibit disrupted functional integration between the DAN and DMN, and the anticorrelation between DMN and DAN would be weakened or even inverted, and that the disruption between the two networks would be significantly associated with attentional deficits.

## Materials and Methods

### Participants

A total of forty-six patients diagnosed with T2DM and 50 healthy controls (HCs) were recruited from the Endocrinology Department and the Health Examination Center of Shaanxi Provincial People’s Hospital from May 2018 to July 2020. All of the participants were between the ages of 45 and 70 years, right-handed, and had at least 6 years of education. T2DM was defined according to the criteria proposed by the American Diabetes Association in 2014, and all patients were self-monitored closely and on stable therapy (exercise, diet, oral medications, or/and insulin). None of the patients had a history of hypoglycemia (blood glucose <3.9 mmol/L) or hyperglycemia (blood glucose >33.3 mmol/L). The HCs were matched with the T2DM patients with respect to age, sex, and educational level. The HCs were selected according to the following criteria: (1) fasting blood glucose (FBG) level <7.0 mmol/l; (2) glycated hemoglobin (HbA1c) <6.0%; and (3) no indication of MCI (defined as a MoCA score ≥ 26). The exclusion criteria for all the participants were as follows: (1) MRI contraindications; (2) indication of dementia (defined as a MMSE score <24); (3) alcohol or other substance dependence; or (4) a history of a brain lesion, such as tumor or stroke, Parkinson’s disease, epilepsy, major depression, or other neurological or psychiatric disorders.

All participants arrived at the Department for MRI between 6:30 and 7:00 pm after dinner, and controlled their blood glucose on the day of the scan, according to the doctor’s instructions. MRI scans were performed after a structured clinical interview and a series of psychological tests. In order to ensure each participant had a relatively stable level of blood glucose, only one participant was examined per day. The testing process and scan time of the HCs were exactly the same as the T2DM patient. During the scan, all participants kept their eyes closed, were calm, and felt no discomfort. Informed consent forms were signed by all of the participants after a full description of the experimental scheme in the study was provided to them. The study has been approved by the Ethics Committee of Shaanxi Provincial People’s Hospital.

### Clinical and Neuropsychological Data

We recorded the patient’s medical history and medications in detail, using a standardized questionnaire, then measured and recorded their weight, height, and blood pressure, and calculated their body mass index (BMI). In addition, laboratory examinations, such as glycated hemoglobin (HbA1c), fasting blood glucose (FBG) concentration, triglyceride (TG) concentration, total cholesterol (TC) concentration, and low-density lipoprotein cholesterol (LDL-C) concentration were performed with standard tests. All the participants underwent a series of detailed neuropsychological tests, which covered multiple cognitive domains. The MMSE was used to assess the overall level of cognition. The MoCA was used to evaluate more detailed general cognition. Attention and psychomotor speed were assessed *via* the Trail-Making Test part A (TMT-A), and the Clock-Drawing Test (CDT) was used to evaluate several relevant cognitive domains, including visual spatial function and execution ability. All of the tests took approximately 30 min to complete. Details of the complications and therapeutic agents for T2DM are provided in Supplementary Tables 1, 2.

### Image Acquisition

All the MR images were acquired at the Department of MRI of Shaanxi Provincial People’s Hospital *via* a Philips 3.0-Tesla scanner with a 16-channel phased-array head coil. Resting-state functional BOLD images were obtained using a gradient-echo planar sequence (TR = 2000 ms, TE = 30 ms, slices = 34, thickness = 4 mm, gap = 0 mm, FOV = 230 mm × 230 mm, matrix = 128 × 128, FA = 90°, and 200 volumes). Structural images were obtained using a sagittal 3-dimensional T1-weighted sequence (TR = 7.5 ms, TE = 3.5 ms, FA = 8°, FOV = 250 mm × 250 mm, matrix = 256 × 256, slice thickness = 1 mm, no gap, and 328 sagittal slices). Fluid-attenuated inversion recovery (FLAIR) and T2-weighted images were acquired to measure visible brain lesions. During the scanning, all of the participants were in a head-first position with their head snugly fixed by straps and foam pads to keep their heads still, and foam padding was used to reduce the impact of noise. All participants were instructed to keep their eyes closed, but not sleep, and to think of nothing in particular.

### Preprocessing of Resting-State Functional Magnetic Resonance Imaging Data

GRETNA^1^ was used to preprocess the functional image data for further analysis in the following steps. First, we removed the first 10 volumes to allow the participants to adapt to the magnetic field, and the processing included slice timing to correct for inter-slice time delays within each volume, within-participant interscan realignment to correct for possible head motion, spatial normalization to a standard brain template in the Montreal Neurological Institute coordinate space using a standard EPI template, resampling into a voxel size of 3 × 3 × 3 mm, and smoothing with an 6-mm FWHM Gaussian kernel.

### Identification of Resting-State Networks

We entered preprocessed resting-state functional magnetic resonance imaging (fMRI) data to implement the spatial group ICA and identify RSNs by the GIFT toolbox^2^. The toolbox performed the analysis as follows: (1) performed principal component analysis to reduce the data of each participants, (2) applied the ICA algorithm, and (3) did back reconstruction for each participant and converted the data into calibrated resting-state FC maps. In this study, we performed GICA 100 times and the number of independent components (maps and corresponding time courses) estimated automatically for each participant was set to 31. In these 31 maps, the best matched 3 components of DAN or DMN were identified by inspecting the aggregate spatial maps and average power spectra with four viewers based on previous resting-state fMRI studies.

To assess small-vessel disease, a single-blind method was used to evaluate the white matter hyperintensity (WMH) and lacunar infarcts on FLAIR images; participants with a rating score >2 were excluded because they could significantly influence the results of the study. Five participants were excluded due to head motion (one patient with T2DM and one HC) and small-vessel disease (one patient with T2DM and two HCs). Finally, a total of 44 patients with T2DM and 47 HCs were enrolled in the study.

### Statistical Analysis

#### Demographic and Clinical Variable Analysis

We used SPSS 18.0 to analyze differences between the T2DM patients and the HCs on the demographic and clinical data and the neuropsychological scores. The independent sample *t*-test (two-tailed) was used to compare group differences on continuous variables, and the chi-square (χ*^2^*) test was used for proportions; *P* < 0.05 was considered to be statistically significant.

#### Intra-Network Functional Connectivity Analysis

The best-matched RSNs of the DAN (IC24) and DMN (IC13 + 29) were selected as the focus of further analysis through visual inspection among the 31 components arising from ICA. First, a one-sample *t*-test was performed to obtain a z-map of each group as the mask (*P* < 0.05), after correcting for multiple comparisons by the False Discovery Rate (FDR). Second, we combined each mask of the HC group and T2DM group into a total mask of each component. Then, a two-sample *t*-test of voxels restricted within the combined mask was used to compare the z-maps of the RSN between the two groups (for the FDR correction, the voxel *P*-value was set to 0.001, and the cluster *P*-value was set to 0.05), and the statistical analyses were performed with age, gender, and educational level as nuisance covariates to control for their potential influence.

#### Inter-Network Functional Connectivity Analysis

The individual level time courses of the selected ICs were obtained by the spatiotemporal double regression method after ICA. Before the FNC analysis, a temporal band-pass filter (ranging from 0.00 to 0.15 Hz) was applied in order to reduce the effects of high-frequency physiological noise, and calculate the correlations between the two IC’s time courses of each participant. Then, the FNC was obtained by calculating the Pearson correlation coefficients of the time courses. Finally, with age, gender, and educational level as covariables, a general linear model (GLM) was used to analyze which pairs of ICs were significantly different between the two groups (for the FDR correction, the voxel *P*-value was set to 0.001, and the cluster *P*-value was set to 0.05).

#### Correlation Analysis

For the intra-network FC, the coordinates of the brain regions with a significant difference in the two-sample *t*-test were extracted to obtain their mean z-scores, and the mean z-scores were used to assess the correlation. The inter-network FCs were estimated as patient correlation coefficients between pairs of time courses of the functional networks, and Fisher’s transformation was used to transform the correlations into z-scores to improve the normality of the correlations. Partial correlation analyses between the abnormal connectivity regions were performed on the clinical data as well as the neuropsychological assessment scores to further explore the relationship between connectivity anomalies and cognitive impairment in patients. The covariates were consistent with the FC analysis. All statistical analyses were performed using SPSS 18.0.

## Results

### Clinical and Neuropsychological Data

The clinical characteristics and neuropsychological data of the patients with T2DM and the HCs are summarized in Table 1. No significant group differences were observed for age, sex, educational level, BMI, blood pressure, or the TG, TC, LDL-C, and CDT scores (all *P*s > 0.05). As expected, the patients exhibited significantly higher levels of HbA1c and FBG than the HCs did (all *P*s < 0.01). The patients also performed significantly worse on the neuropsychological assessments: the MoCA (*P* < 0.01) and the TMT-A (*P* < 0.05).

**TABLE 1:** Demographic, clinical, and cognitive data of the patients with T2DM and the HCs.

Variable	T2DM (*n* = 44)	HCs (*n* = 47)	*P*-value
Age (years)	55.2 ± 7.26	54.28 ± 6.90	0.53
Male/female	26/18	30/17	0.64^#^
Educational level (years)	13.34 ± 2.79	13.98 ± 3.00	0.30
Diabetes duration (years)	10.27 ± 5.57	–	–
Systolic BP (mmHg)	125.25 ± 13.84	125.87 ± 8.39	0.79
Diastolic BP (mmHg)	80.57 ± 7.57	80.55 ± 6.51	0.99
BMI (kg/m^2^)	24.26 ± 2.69	23.88 ± 2.80	0.51
FBG (mmol/L)	8.14 ± 2.29	5.11 ± 0.60	<0.01
HbA1c (%)	7.79 ± 1.58	5.46 ± 0.41	<0.01
TG (mmol/L)	1.74 ± 0.76	1.89 ± 1.20	0.47
TC (mmol/L)	4.76 ± 1.52	4.80 ± 0.97	0.87
LDL-C (mmol/L)	2.61 ± 0.75	2.84 ± 0.96	0.22
MMSE	28.30 ± 1.70	28.39 ± 1.51	0.78
MoCA	25.31 ± 3.02	27.17 ± 1.11	<0.01
TMT-A	85.23 ± 33.14	69.57 ± 32.20	0.03
CDT	20.44 ± 8.43	22.69 ± 6.50	0.16

*Normally distributed variables are presented as mean ± standard deviation. BMI, body mass index; FBG, fasting blood glucose; TG, triglycerides; TC, total cholesterol; LDL, low-density lipoprotein; HbA1c, glycated hemoglobin; MMSE, Mini-Mental State Examination; MoCA, Montreal Cognitive Assessment; TMT-A, Trail Making Test A; CDT, Clock Drawing Test.*

*^#^P for the χ^2^ test.*

### Resting-State Networks

**FIGURE 1:**
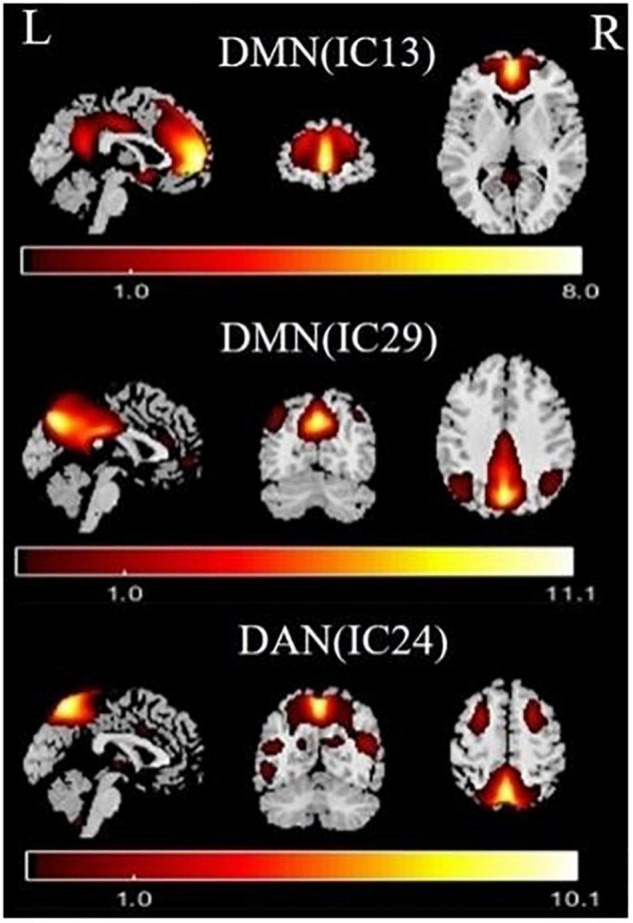
Functional relevant resting-state networks (RSNs). The spatial maps of three independent components (ICs) were selected as the RSNs for further analysis. DMN, default mode network; DAN, dorsal attention network.

Independent component analysis identified the DMN and DAN (Figure 1). The DMN (IC13 + IC29) mainly included the medial prefrontal cortex, the posterior cingulate cortex/precuneus, and the bilateral inferior parietal cortex. The DAN (IC24) mainly included the bilateral intraparietal sulcus, frontal eye field, and ventral parietal cortex.

### Altered Intra-/Inter-Network Functional Connectivity

**FIGURE 2:**
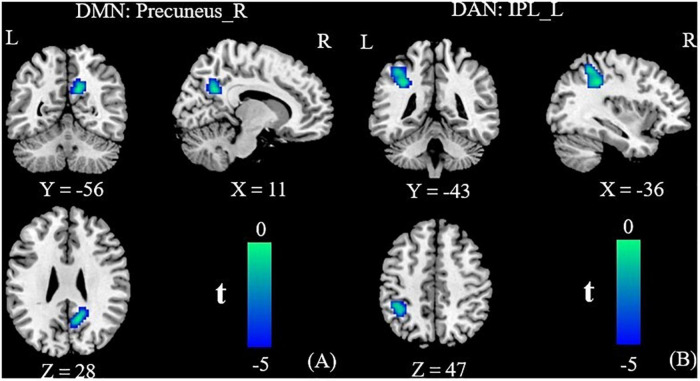
Group functional connectivity (FC) differences within RSNs. Significant differences between the T2DM and HC groups were found within the DMN **(A)** and the DAN **(B)**. DMN, default mode network; DAN, dorsal attention network; IPL, inferior parietal lobule; R, right; L, left.

**FIGURE 3:**
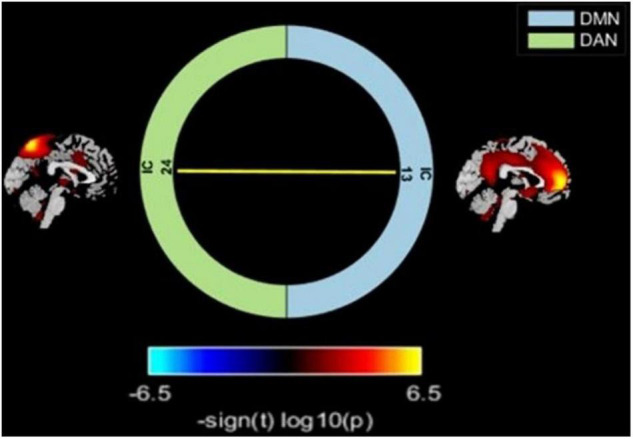
Comparisons of inter-network FC alterations between the RSNs in the T2DM and HC groups. T2DM group exhibited increased FC between the DAN and DMN compared with HC. Color scale denotes the *t*-value. Warm color represents positive functional connectivity; cold color represents negative functional connectivity.

**FIGURE 4:**
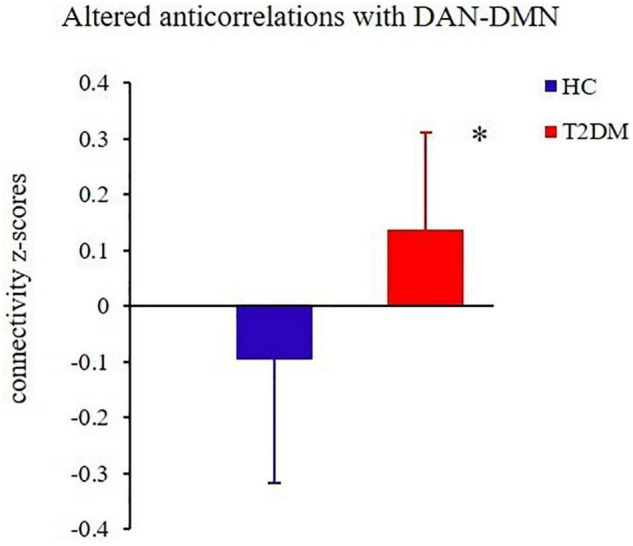
The groups differed significantly in their average connectivity between IC24 and IC13 in the DAN and DMN. The horizontal axis represents the groups, the ordinate axis represents average functional connectivity, and the error bars represent standard deviations, *P < 0.05.

Relative to the HC group, the T2DM patients had decreased FC in the right precuneus within the DMN and decreased FC in the left inferior parietal lobule (IPL) within the DAN (Table 2 and Figure 2). Subsequent FNC analysis showed that, compared with the controls, the patients with T2DM exhibited significantly increased inter-network connectivity between the DAN (IC24) and DMN (IC13) (Figure 3). The bar graphs display the average connectivity z-scores between the DAN (IC24) and DMN (IC13) in the HC and T2DM groups, the average connectivity z-score was negative in the HC group and positive in the T2DM group (Figure 4).

**TABLE 2:** Abnormal functional connectivity in the patients with T2DM compared to the HC group.

Resting-state network	Brain region	Peak MNI coordinates			Voxel (mm^3^)	BA	*t*-value
		*X*	*Y*	*Z*			
DMN	R precuneus	12	−54	30	73	31	−5.59
DAN	L inferior parietal lobule	−36	−39	36	107	40	−5.49

*BA, Brodmann’s area; MNI, Montreal Neurological Institute; L, left; R, right. Group differences in functional connectivity were evaluated by two-sample t-tests (for FDR correction, the voxel P-value was set to 0.001, and the cluster P-value was set to 0.05).*

### Correlation Analysis

**FIGURE 5:**
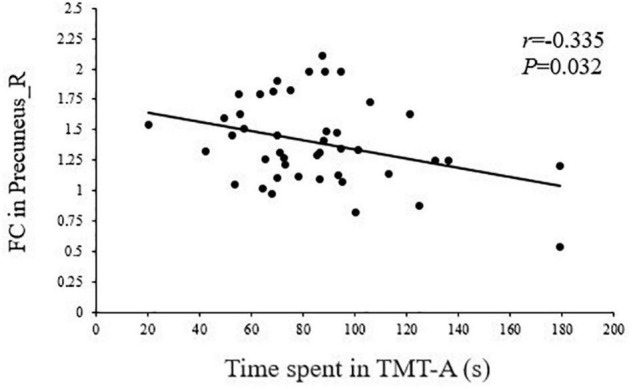
Correlation between TMT-A scores and the right precuneus within the default mode network (DMN) in T2DM patients (*r* = –0.335, *P* = 0.032).

**FIGURE 6:**
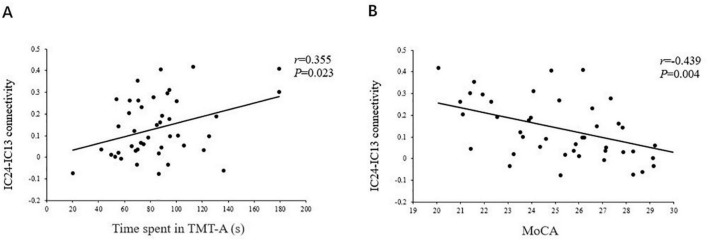
The correlations of the strength of connectivity between the IC24 and IC13 in the DAN and the DMN with the TMT scores (*r* = 0.355, *P* = 0.023) **(A)**, and the MoCA scores (*r* = –0.439, *P* = 0.004) **(B)**.

As shown in Figures 5, 6, the FC of the right precuneus within the DMN was inversely correlated with TMT-A scores (*r* = −0.335, *P* = 0.032) in patients with T2DM, and the connectivity strength between the IC24 and IC13 in the DAN and DMN was significantly correlated with the TMT-A scores (*r* = 0.355, *P* = 0.023) and the MoCA scores (*r* = −0.439, *P* = 0.004).

## Discussion

The ICA analysis in the present study showed that patients with T2DM exhibited abnormal functional integration within the DAN and DMN, compared with the HC group. Using FNC analysis, we further found the patients exhibited significantly increased internetwork connectivity in the DAN-DMN, and that FC disruptions in the two networks were significantly associated with poorer neuropsychological scores. These findings suggest that the FC changes of the DMN and DAN are mainly characterized by a loss of intra-network connectivity and an increase in the connectivity between the networks that normally exhibit anticorrelated activities.

### Altered Functional Connectivity Within the Default Mode Network and Dorsal Attention Network

Consistent with previously reported findings (Cui et al., 2015), our results confirm a significant decrease of DMN in patients with T2DM. The DMN plays an important role in both primary perceived control and advanced cognitive processing. One study has found that a DMN with high metabolic activity increases aerobic glycolysis and makes the hub region more vulnerable to metabolic abnormalities and β-amyloid deposition, resulting in impaired FC (Buckner et al., 2005). The precuneus is the core node of the DMN, and it is also one of the brain regions with the highest spontaneous neural activity and metabolism in the resting-state (Cavanna and Trimble, 2006; Tessitore et al., 2012). Insulin resistance and hyperglycemia in patients with T2DM accelerate β-amyloid deposition (Biessels et al., 2006), which may be the reason for the decreased FC of the precuneus in the current study. As the center of behavioral control, dysfunction of the precuneus is closely related to cognitive impairment (Cavanna and Trimble, 2006). Previous studies have found that precuneus-DMN disconnections may be a marker of AD pathology (Klaassens et al., 2017). Furthermore, the precuneus may process not only spatial attention, but also attention shifts between object features (Nagahama et al., 1999; Cavanna and Trimble, 2006). The TMT-A is highly sensitive to impairments in visual search and attention (Talwar et al., 2020). In this study, the FC of the right precuneus within the DMN was inversely correlated with the TMT-A scores, suggesting that the reduced FC in the precuneus may be related to an attention management disorder among patients with T2DM.

The DAN plays a central role in visuospatial attention and visual motor function, and it participates in top-down processing of attentional orientation to ensure the completion of cognitive tasks (Xia et al., 2015). The IPL is a core region in the DAN, and Clemens et al. (2013) found that during the processing of attention, the IPL is continuously and stably activated in task-fMRI research of HCs, suggesting that the IPL is involved in the regulation and integration of attention. Previous research has found that patients with attention deficit hyperactivity disorder have abnormal activity in the parietal region, which plays a role in top-down attention control (Aboitiz et al., 2014). In addition, abnormal activity of the IPL has been found in patients with depression (Zhou et al., 2010) and AD (Prvulovic et al., 2002; Hao et al., 2005), and it has been suggested that IPL dysfunction is related to the reduced attention ability of patients to extraneous stimuli. Clinical and epidemiological studies have shown that attention is compromised in patients with T2DM, especially selective attention and the ability to maintain attention (Garcia-Casares et al., 2014; Toth, 2014). Consistent with a previous study by Xia et al. (2015), our study also found reduced FC in the IPL in T2DM patients. Considering the central role of the IPL in the DAN, we speculate that abnormal changes in the IPL may be the neural basis of attentional impairment in T2DM patients. In addition, our study found that patients with T2DM spent more time on the TMT-A test, suggesting they have attention and neuromotor speed impairments, which also confirms this hypothesis to a certain extent.

### Altered Anticorrelation Between the Default Mode Network and Dorsal Attention Network

At present, the field of cognitive neuroscience generally believes that the core network subsystem of the DAN and DMN demonstrate an intrinsic anticorrelation in healthy adults, which is thought to be a core feature of the human brain’s intrinsic architecture (Dixon et al., 2017). Fox et al. (2005) suggested that the anticorrelations of the two networks might be interpreted as competition between focused attention and processes subserving stimulus independent thought, which indicates that the networks’ intrinsic functional antagonism supports a range of cognitive functions. In addition, our study found patients with T2DM seem to attenuate this decoupling effect, suggesting decreased inhibition between the core subsystems of the two networks. The reduced anticorrelation between the DAN and DMN is thought to be related to the poor modulation of attentional processes in response to shifting cognitive demands and inefficiency in processing cognitive resources (Wang et al., 2019). This is consistent with the clinical observations that T2DM patients have a significantly prolonged response time to stimulus signals, diminished ability to focus attention, and other cognitive impairments (Cooray et al., 2011). Our study also found that TMT-A and MoCA scores were poorer in patients with T2DM, indicating that they had impairments in attention and other general cognitive function. Furthermore, the strength of connectivity between the DAN and DMN core subsystem in patients with T2DM were negatively correlated with their MoCA scores, and positively correlated with their TMT-A scores. The MoCA covers important cognitive domains, including attention, concentration, and executive control (Nasreddine et al., 2005). Compared with the MMSE, it can detect mild cognitive impairment more sensitively, and the increased time patients spend on the TMT-A test indicates their attention and psychomotor speed is abnormal (Talwar et al., 2020). This further illustrates that an abnormal network interaction between the DAN and DMN may be the neural basis of attention and general cognitive dysfunction in T2DM. Therefore, we speculate that the change in the functional pattern of this interactive network inversion may become a neuroimaging marker for detecting cognitive impairment in patients with T2DM and monitoring the results of intervention. Moreover, a study has demonstrated that cognitive training can reverse the inverted functional connection (Cao et al., 2016), which also provides a worthwhile intervention for treatment of T2DM cognitive impairment.

Recent reports revealed that the salience network (SN) tightly regulates the antagonistic DAN and DMN for successful fulfillment of specific tasks and/or maintenance of certain behavioral state (Gao and Lin, 2012; Zhu et al., 2016). Integrity of the SN is necessary for the efficient regulation of flexibly coupling either with the DAN or DMN networks during different brain states, and that a failure of this regulation leads to attentional lapses and other inefficient cognitive control (Bonnelle et al., 2012). Our previous studies have shown that there are abnormal changes in the SN of patients with T2DM under different cognitive stages, the right frontoinsular cortex (the core region of the SN) may be a useful imaging biomarker for supplementary assessment of early cognitive dysfunction in patients with T2DM (Zhang et al., 2021). This study found the anti-correlation between DAN and DMN showed disease-related disruptions in patients with T2DM. However, whether the deficits in the balance between the DAN and DMN is related to impaired “regulating” role of the SN, future studies are needed to independently validate this finding and test this hypothesis.

## Limitations

This study has some limitations. First, a cross-sectional design was used in this study, and the small sample size precluded the ability to use a better stratified research design. Second, the medications of the patients with T2DM were not the same, and medications may have had an effect on neural activity. Third, the cognitive screening instruments we chose were relatively simple, which may affect us to fully reveal whether the destruction of anti-correlation between DAN and DMN is related to different aspects of attention or other related cognitive dysfunctions in this study. Finally, this study used the data-driven ICA method to explore abnormal changes in the FC of the interactive networks in T2DM. Future research should use causal analysis and other methods to evaluate the specificity and directionality of functional coupling between these brain networks, in order to provide richer neuroimaging evidence to elucidate the mechanisms underlying cognitive impairments in patients with T2DM.

## Conclusion

To our knowledge, this study first discovered that the inherent neural mechanism between the DAN and DMN was eliminated in patients with T2DM, and the abnormal changes in FC between the two networks were related to attention and general cognitive function. These findings indicate that abnormal DAN-DMN interactions may be the neural basis of T2DM-related cognitive deficits.

## Data Availability Statement

The original contributions presented in the study are included in the article/Supplementary Material, further inquiries can be directed to the corresponding author/s.

## Ethics Statement

The studies involving human participants were reviewed and approved by the Ethics Committee of Shaanxi Provincial People’s Hospital. The patients/participants provided their written informed consent to participate in this study.

## Author Contributions

YL and DZ drafted the manuscript and designed the study. YL performed the statistical analysis. FQ and JG contributed to conducting the study and revised the manuscript. MT and KA provided technical support. XY, XL, ZS, and YS collected the data. XZ helped to design the study and revised the manuscript. All authors contributed to the article and approved the submitted version.

## Conflict of Interest

KA was employed by the company Philips Healthcare. The remaining authors declare that the research was conducted in the absence of any commercial or financial relationships that could be construed as a potential conflict of interest.

## Publisher’s Note

All claims expressed in this article are solely those of the authors and do not necessarily represent those of their affiliated organizations, or those of the publisher, the editors and the reviewers. Any product that may be evaluated in this article, or claim that may be made by its manufacturer, is not guaranteed or endorsed by the publisher.

## Footnotes

1
https://www.nitrc.org/projects/gretna/

2
mialab.mrn.org/software/gift/

## References

[B1] Aboitiz F. Ossandon T. Zamorano F. Palma B. Carrasco X. (2014). Irrelevant stimulus processing in ADHD: catecholamine dynamics and attentional networks. *Front. Psychol.* 5:183. 10.3389/fpsyg.2014.00183 24723897 PMC3972460

[B2] Baggio H. C. Segura B. Sala-Llonch R. Marti M. J. Valldeoriola F. Compta Y. (2015). Cognitive impairment and resting-state network connectivity in Parkinson’s disease. *Hum. Brain Mapp.* 36 199–212. 10.1002/hbm.22622 25164875 PMC6869118

[B3] Bedse G. Di Domenico F. Serviddio G. Cassano T. (2015). Aberrant insulin signaling in Alzheimer’s disease: current knowledge. *Front. Neurosci.* 9:204. 10.3389/fnins.2015.00204 26136647 PMC4468388

[B4] Biessels G. J. De Leeuw F. E. Lindeboom J. Barkhof F. Scheltens P. (2006). Increased cortical atrophy in patients with Alzheimer’s disease and type 2 diabetes mellitus. *J. Neurol Neurosurg. Psychiatry* 77 304–307. 10.1136/jnnp.2005.069583 16484636 PMC2077690

[B5] Bonnelle V. Ham T. E. Leech R. Kinnunen K. M. Mehta M. A. Greenwood R. J. (2012). Salience network integrity predicts default mode network function after traumatic brain injury. *Proc. Natl. Acad. Sci. U S A.* 109 4690–4695. 10.1073/pnas.1113455109 22393019 PMC3311356

[B6] Buckner R. L. Snyder A. Z. Shannon B. J. LaRossa G. Sachs R. Fotenos A. F. (2005). Molecular, structural, and functional characterization of Alzheimer’s disease: evidence for a relationship between default activity, amyloid, and memory. *J. Neurosci.* 25 7709–7717. 10.1523/JNEUROSCI.2177-05.2005 16120771 PMC6725245

[B7] Burk J. A. Blumenthal S. A. Maness E. B. (2018). Neuropharmacology of attention. *Eur. J. Pharmacol.* 835 162–168. 10.1016/j.ejphar.2018.08.008 30092180 PMC6140347

[B8] Cao W. Cao X. Hou C. Li T. Cheng Y. Jiang L. (2016). Effects of Cognitive Training on Resting-State Functional Connectivity of Default Mode, Salience, and Central Executive Networks. *Front. Aging Neurosci.* 8:70. 10.3389/fnagi.2016.00070 27148042 PMC4828428

[B9] Cavanna A. E. Trimble M. R. (2006). The precuneus: a review of its functional anatomy and behavioural correlates. *Brain* 129 564–583. 10.1093/brain/awl004 16399806

[B10] Clemens B. Zvyagintsev M. Sack A. T. Heinecke A. Willmes K. Sturm W. (2013). Comparison of fMRI activation patterns for test and training procedures of alertness and focused attention. *Restor. Neurol Neurosci.* 31 311–336. 10.3233/RNN-120266 23403596

[B11] Cooray G. Nilsson E. Wahlin A. Laukka E. J. Brismar K. Brismar T. (2011). Effects of intensified metabolic control on CNS function in type 2 diabetes. *Psychoneuroendocrinology* 36 77–86. 10.1016/j.psyneuen.2010.06.009 20656408

[B12] Cui Y. Jiao Y. Chen H. J. Ding J. Luo B. Peng C. Y. (2015). Aberrant functional connectivity of default-mode network in type 2 diabetes patients. *Eur. Radiol.* 25 3238–3246. 10.1007/s00330-015-3746-8 25903712 PMC4595523

[B13] Delbeuck X. Collette F. Van der Linden M. (2007). Is Alzheimer’s disease a disconnection syndrome? Evidence from a crossmodal audio-visual illusory experiment. *Neuropsychologia* 45 3315–3323. 10.1016/j.neuropsychologia.2007.05.001 17765932

[B14] Delbeuck X. Van der Linden M. Collette F. (2003). Alzheimer’s disease as a disconnection syndrome? *Neuropsychol. Rev.* 13 79–92. 10.1023/a:1023832305702 12887040

[B15] Dixon M. L. Andrews-Hanna J. R. Spreng R. N. Irving Z. C. Mills C. Girn M. (2017). Interactions between the default network and dorsal attention network vary across default subsystems, time, and cognitive states. *Neuroimage* 147 632–649. 10.1016/j.neuroimage.2016.12.073 28040543

[B16] Egner S. Reimann S. Hoeger R. Zangemeister W. H. (2018). Attention and Information Acquisition: Comparison of Mouse-Click with Eye-Movement Attention Tracking. *J. Eye Mov. Res.* 11:4. 10.16910/jemr.11.6.4 33828714 PMC7908465

[B17] Fox M. D. Raichle M. E. (2007). Spontaneous fluctuations in brain activity observed with functional magnetic resonance imaging. *Nat. Rev. Neurosci.* 8 700–711. 10.1038/nrn2201 17704812

[B18] Fox M. D. Snyder A. Z. Vincent J. L. Corbetta M. Van Essen D. C. Raichle M. E. (2005). The human brain is intrinsically organized into dynamic, anticorrelated functional networks. *Proc. Natl. Acad. Sci. U S A.* 102 9673–9678. 10.1073/pnas.0504136102 15976020 PMC1157105

[B19] Gao W. Lin W. (2012). Frontal parietal control network regulates the anti-correlated default and dorsal attention networks. *Hum. Brain Mapp.* 33 192–202. 10.1002/hbm.21204 21391263 PMC3131466

[B20] Garcia-Casares N. Jorge R. E. Garcia-Arnes J. A. Acion L. Berthier M. L. Gonzalez-Alegre P. (2014). Cognitive dysfunctions in middle-aged type 2 diabetic patients and neuroimaging correlations: a cross-sectional study. *J. Alzheimers Dis.* 42 1337–1346. 10.3233/JAD-140702 25024335

[B21] Gibas K. J. (2017). The starving brain: Overfed meets undernourished in the pathology of mild cognitive impairment (MCI) and Alzheimer’s disease (AD). *Neurochem. Int.* 110 57–68. 10.1016/j.neuint.2017.09.004 28899812

[B22] Hao J. Li K. Li K. Zhang D. Wang W. Yang Y. (2005). Visual attention deficits in Alzheimer’s disease: an fMRI study. *Neurosci. Lett.* 385 18–23. 10.1016/j.neulet.2005.05.028 15970381

[B23] Karvani M. Simos P. Stavrakaki S. Kapoukranidou D. (2019). Neurocognitive impairment in type 2 diabetes mellitus. *Hormones* 18 523–534. 10.1007/s42000-019-00128-2 31522366

[B24] Katsuki F. Constantinidis C. (2014). Bottom-up and top-down attention: different processes and overlapping neural systems. *Neuroscientist* 20 509–521. 10.1177/1073858413514136 24362813

[B25] Kelly A. M. Uddin L. Q. Biswal B. B. Castellanos F. X. Milham M. P. (2008). Competition between functional brain networks mediates behavioral variability. *Neuroimage* 39 527–537. 10.1016/j.neuroimage.2007.08.008 17919929

[B26] Klaassens B. L. van Gerven J. M. A. van der Grond J. de Vos F. Moller C. Rombouts S. (2017). Diminished Posterior Precuneus Connectivity with the Default Mode Network Differentiates Normal Aging from Alzheimer’s Disease. *Front. Aging Neurosci.* 9:97. 10.3389/fnagi.2017.00097 28469571 PMC5395570

[B27] Musen G. Jacobson A. M. Bolo N. R. Simonson D. C. Shenton M. E. McCartney R. L. (2012). Resting-state brain functional connectivity is altered in type 2 diabetes. *Diabetes* 61 2375–2379. 10.2337/db11-1669 22664957 PMC3425418

[B28] Nagahama Y. Okada T. Katsumi Y. Hayashi T. Yamauchi H. Sawamoto N. (1999). Transient neural activity in the medial superior frontal gyrus and precuneus time locked with attention shift between object features. *Neuroimage* 10 193–199. 10.1006/nimg.1999.0451 10417251

[B29] Nasreddine Z. S. Phillips N. A. Bedirian V. Charbonneau S. Whitehead V. Collin I. (2005). The Montreal Cognitive Assessment, MoCA: a brief screening tool for mild cognitive impairment. *J. Am. Geriatr. Soc.* 53 695–699. 10.1111/j.1532-5415.2005.53221.x 15817019

[B30] Owens M. M. Yuan D. Hahn S. Albaugh M. Allgaier N. Chaarani B. (2020). Investigation of Psychiatric and Neuropsychological Correlates of Default Mode Network and Dorsal Attention Network Anticorrelation in Children. *Cereb. Cortex* 30 6083–6096. 10.1093/cercor/bhaa143 32591777 PMC8086768

[B31] Perry R. J. Watson P. Hodges J. R. (2000). The nature and staging of attention dysfunction in early (minimal and mild) Alzheimer’s disease: relationship to episodic and semantic memory impairment. *Neuropsychologia* 38 252–271. 10.1016/s0028-3932(99)00079-2 10678692

[B32] Prvulovic D. Hubl D. Sack A. T. Melillo L. Maurer K. Frolich L. (2002). Functional imaging of visuospatial processing in Alzheimer’s disease. *Neuroimage* 17 1403–1414. 10.1006/nimg.2002.1271 12414280

[B33] Qian S. Zhang J. Yan S. Shi Z. Wang Z. Zhou Y. (2020). Disrupted Anti-correlation Between the Default and Dorsal Attention Networks During Hyperthermia Exposure: An fMRI Study. *Front. Hum. Neurosci.* 14:564272. 10.3389/fnhum.2020.564272 33304249 PMC7693425

[B34] Song Z. Chen J. Wen Z. Zhang L. (2021). Abnormal functional connectivity and effective connectivity between the default mode network and attention networks in patients with alcohol-use disorder. *Acta Radiol.* 62 251–259. 10.1177/0284185120923270 32423229

[B35] Talwar N. Churchill N. W. Hird M. A. Tam F. Graham S. J. Schweizer T. A. (2020). Functional magnetic resonance imaging of the trail-making test in older adults. *PLoS One* 15:e0232469. 10.1371/journal.pone.0232469 32396540 PMC7217471

[B36] Tessitore A. Esposito F. Vitale C. Santangelo G. Amboni M. Russo A. (2012). Default-mode network connectivity in cognitively unimpaired patients with Parkinson disease. *Neurology* 79 2226–2232. 10.1212/WNL.0b013e31827689d6 23100395

[B37] Toth C. (2014). Diabetes and neurodegeneration in the brain. *Handb. Clin. Neurol.* 126 489–511. 10.1016/B978-0-444-53480-4.00035-7 25410241

[B38] Van Dam N. T. Sano M. Mitsis E. M. Grossman H. T. Gu X. (2013). Functional neural correlates of attentional deficits in amnestic mild cognitive impairment. *PLoS One* 8:e54035. 10.1371/journal.pone.0054035 23326568 PMC3543395

[B39] Verdile G. Fuller S. J. Martins R. N. (2015). The role of type 2 diabetes in neurodegeneration. *Neurobiol. Dis.* 84 22–38. 10.1016/j.nbd.2015.04.008 25926349

[B40] Wang J. Liu J. Wang Z. Sun P. Li K. Liang P. (2019). Dysfunctional interactions between the default mode network and the dorsal attention network in subtypes of amnestic mild cognitive impairment. *Aging* 11 9147–9166. 10.18632/aging.102380 31645482 PMC6834429

[B41] Whitfield-Gabrieli S. Ford J. M. (2012). Default mode network activity and connectivity in psychopathology. *Annu. Rev. Clin. Psychol.* 8 49–76. 10.1146/annurev-clinpsy-032511-143049 22224834

[B42] Xia W. Wang S. Rao H. Spaeth A. M. Wang P. Yang Y. (2015). Disrupted resting-state attentional networks in T2DM patients. *Sci. Rep.* 5:11148. 10.1038/srep11148 26053355 PMC4459168

[B43] Xing C. Zhang J. Cui J. Yong W. Hu J. Yin X. (2020). Disrupted Functional Network Connectivity Predicts Cognitive Impairment in Presbycusis Patients. *Front. Aging Neurosci.* 12:246. 10.3389/fnagi.2020.00246 32903748 PMC7438913

[B44] Yang S. Q. Xu Z. P. Xiong Y. Zhan Y. F. Guo L. Y. Zhang S. (2016). Altered Intranetwork and Internetwork Functional Connectivity in Type 2 Diabetes Mellitus With and Without Cognitive Impairment. *Sci. Rep.* 6:32980. 10.1038/srep32980 27622870 PMC5020685

[B45] Zhang D. Lei Y. Gao J. Qi F. Yan X. Ai K. (2021). Right Frontoinsular Cortex: A Potential Imaging Biomarker to Evaluate T2DM-Induced Cognitive Impairment. *Front. Aging Neurosci.* 13:674288. 10.3389/fnagi.2021.674288 34122050 PMC8193040

[B46] Zhou X. Zhang J. Chen Y. Ma T. Wang Y. Wang J. (2014). Aggravated cognitive and brain functional impairment in mild cognitive impairment patients with type 2 diabetes: a resting-state functional MRI study. *J. Alzheimers Dis.* 41 925–935. 10.3233/JAD-132354 24705547

[B47] Zhou Y. Yu C. Zheng H. Liu Y. Song M. Qin W. (2010). Increased neural resources recruitment in the intrinsic organization in major depression. *J. Affect. Disord.* 121 220–230. 10.1016/j.jad.2009.05.029 19541369

[B48] Zhu D. C. Majumdar S. Korolev I. O. Berger K. L. Bozoki A. C. (2013). Alzheimer’s disease and amnestic mild cognitive impairment weaken connections within the default-mode network: a multi-modal imaging study. *J. Alzheimers Dis.* 34 969–984. 10.3233/JAD-121879 23313926

[B49] Zhu H. Zhou P. Alcauter S. Chen Y. Cao H. Tian M. (2016). Changes of intranetwork and internetwork functional connectivity in Alzheimer’s disease and mild cognitive impairment. *J. Neural Eng.* 13:046008. 10.1088/1741-2560/13/4/046008 27247279

