# Supplementary material for: Dysfunctional Interaction Between the Dorsal Attention Network and the Default Mode Network in Patients With Type 2 Diabetes Mellitus
Source: Front Hum Neurosci. 2021 Dec 24;15:796386. doi: 10.3389/fnhum.2021.796386 (PMC8741406; doi:10.3389/fnhum.2021.796386)
Supplement: Supplementary file 1 [file Table_1.DOCX]

**Supplementary Table 1.** T2DM complications

| Complication | Number of patients |
| --- | --- |
| No complications | 27 |
| Nephropathy | 5 |
| Peripheral neuropathy | 5 |
| Retinopathy | 3 |
| Nephropathy + peripheral neuropathy | 4 |
