# Supplementary material for: Dysfunctional Interaction Between the Dorsal Attention Network and the Default Mode Network in Patients With Type 2 Diabetes Mellitus
Source: Front Hum Neurosci. 2021 Dec 24;15:796386. doi: 10.3389/fnhum.2021.796386 (PMC8741406; doi:10.3389/fnhum.2021.796386)
Supplement: Supplementary file 2 [file Table_2.DOCX]

**Supplementary Table 2.** T2DM therapeutic agents

| Therapeutic agent | Medication | Number of patients |
| --- | --- | --- |
| Dietary restriction |  | 5 |
| Insulin |  | 4 |
|  | Metformin | 10 |
|  | Sulfonylureas | 2 |
|  | Acarbose | 4 |
| Oral medication | Metformin + sulfonylureas | 4 |
|  | Metformin + Acarbose | 5 |
|  | Metformin + Acarbose+ sulfonylureas | 4 |
|  | Metformin | 2 |
| Insulin+oral medication | Acarbose | 2 |
|  | Metformin + acarbose | 1 |
|  | Metformin + sulfonylureas | 1 |
